# The acute effects of targeted abdominal muscle activation training on spine stability and neuromuscular control

**DOI:** 10.1186/s12984-016-0126-9

**Published:** 2016-02-27

**Authors:** Daniel J. Southwell, Nicole F. Hills, Linda McLean, Ryan B. Graham

**Affiliations:** School of Physical and Health Education, Nipissing University, 100 College Drive, Box 5002, North Bay, ON P1B 8L7 Canada; School of Rehabilitation Therapy, Queen’s University, 31 George Street, Kingston, ON K7L 3N6 Canada; School of Rehabilitation Sciences, University of Ottawa, 451 Smyth Road, Ottawa, ON K1H 8M5 Canada; School of Human Kinetics, University of Ottawa, 125 University Private, Ottawa, ON K1N 6N5 Canada

**Keywords:** *Spine*, *Stability*, *Rehabilitation*, *Transversus abdominis*, *Physiotherapy*, *Electromyography*, *Dynamical systems*, *Neuromuscular*

## Abstract

**Background:**

Targeted activation of the transversus abdominis (TrA) muscle through the abdominal drawing-in maneuver (ADIM) is a frequently prescribed exercise for the prevention and rehabilitation of low back pain. However, there is still debate over the role the ADIM plays in maintaining a stable spine during movement. Thus, a single cohort pre/post-intervention protocol was used to examine whether 5 min of ADIM training prior to a dynamic movement task alters dynamic spine stability and control.

**Methods:**

Thirteen healthy participants performed a repetitive spine flexion task twice, once before and once after they received biofeedback training on how to correctly perform the ADIM in standing. Abdominal and back muscle activation (indwelling and surface electromyography, EMG) and 3D kinematic data were recorded during all trials. EMG activation (percent maximum) and local dynamic stability of spine movement [maximum finite-time Lyapunov exponent (λ_max_)] were compared before and after the training using Friedman’s rank test and repeated-measures ANOVA, respectively. To assess the moderating effects of absolute changes in EMG (∆EMG) of each muscle after training on changes in stability, the ∆EMG (peak and mean) were added to the ANOVA as separate covariates (ANCOVA).

**Results:**

Following ADIM training, there were greater peak and mean levels of activation in all tested abdominal muscles, including TrA, (*p* < 0.05), but not in the back muscles. The ANOVA showed no significant change in λ_max_ following training (*p* = 0.633). However, after considering the moderating effects of the ∆EMG seen in each muscle with training, it was found that only changes in TrA EMG significantly influenced stability. The ANCOVA revealed a significant main effect of training on stability as well as a significant interaction effect between training and ∆EMG recorded from TrA (*p* < 0.05); those with larger increases in TrA activation demonstrated larger improvements in stability.

**Conclusion:**

As a group, 5 min of ADIM training did not change spine stability during dynamic movement. However, those who were most successful in improving TrA activation with a 5-min ADIM training session showed the greatest improvements in local dynamic spine stability after training. As such, dynamic spine stability in some individuals may benefit from ADIM training.

**Electronic supplementary material:**

The online version of this article (doi:10.1186/s12984-016-0126-9) contains supplementary material, which is available to authorized users.

## Background

The osteoligamentous spine is naturally unstable, with the lumbar spine buckling under loads as little as 90 N (~9Kg) [1]. The significance of the trunk musculature in providing stability to the lumbar spine is well established [1]; however, there is still much debate regarding the role and relative importance of specific trunk muscles [2–5]. Some researchers argue that no single trunk muscle is more important to the stability of the spine than any other, but rather stability comes from the combined action of all the trunk muscles [2–4]. Alternate views have suggested that the transversus abdominis (TrA) is critical to the stability of the spine due to its horizontal fibre orientation [5].

Bilateral activation of the TrA muscle can contribute tension to the fascial structures of the lumbar region [6], can modulate intra-abdominal pressure [7–9], and can compress the sacroiliac joint [10]. Moreover, Hodges et al. [8] induced low back pain (LBP) in otherwise healthy individuals via intramuscular injection of hypertonic saline, and noted that the experimentally induced pain altered feed-forward recruitment of the TrA. The TrA muscle consistently demonstrated delayed onset or reduced electromyography (EMG) amplitude while the participants were experiencing pain [8]. This onset delay was not observed in the other trunk muscles tested (i.e. erector spinae, deep multifidus, external oblique, and internal oblique) [8]. Interestingly, the delayed onset of the TrA persisted at a follow-up evaluation despite the pain intensity having subsided to a minimal level, which suggests that, once affected by acute pain, motor recruitment patterns may remain altered after pain has subsided [8]. These research contributions have been used to support the clinical practice of training TrA motor control in patients with LBP [6, 8, 11, 12].

Maneuvers, such as the abdominal drawing-in maneuver (ADIM), which is thought to preferentially activate the TrA, are commonly used in physical therapy treatment plans for the management of LBP. Typically in clinical practice, the ADIM is taught in a static position with or without biofeedback. It is then recommended that patients perform the ADIM prior to and during dynamic tasks under the clinical assumption that preferentially activating the TrA will improve spine stability [10]. However, a debate appears to exist in the literature regarding the effectiveness of the use of the ADIM to maintain a stable spine [13, 14]. Evidence in the literature suggests that there is little benefit to using an ADIM to increase mechanical stability of the spine, and that bracing activities are more effective in enhancing stability [13]. Therefore, the goal of this study was to test the clinical assumption that training healthy participants to preferentially activate their TrA using the ADIM improves spine stability during dynamic movement.

Dynamic spine stability is defined as the ability of the spine to follow or return to an intended trajectory during a movement task [15]. Dynamic stability is not accurately tested in most biomechanical models as few include contributions of intra-abdominal pressure and fascial tension [14]. One way to assess control and take into account all aspects of spine stability is to look directly at the outcome kinematics during repetitive movement using non-linear dynamical systems analyses [16]. Recent work has shown that local dynamic stability analysis exhibits good discriminative ability in predicting fall risk [17, 18]. Furthermore, it has recently been shown that local dynamic stability can be impaired with acute experimentally-induced LBP [19]. Due to this specificity in the ability to quantify dynamic stability, non-linear dynamical systems analysis offers a potential method to assess the impact of the ADIM on stability.

The purposes of this study were: i) to examine whether TrA activation was increased during a repetitive dynamic trunk movement task following 5 min of ADIM training using ultrasound biofeedback, ii) to examine whether local dynamic spine stability (λ_max_) was increased during a repetitive dynamic trunk movement task following the same training, and (iii) to assess whether changes in TrA activation were associated with changes in local dynamic spine stability. It was hypothesized that, following ADIM training, participants would show an increased level of activation of the TrA, which consequently would lead to an increase in dynamic spine stability (lower λ_max_).

## Methods

### Participants

Thirteen healthy students (7 M, 6 F) with no history of LBP or current musculoskeletal/neurological injuries were recruited through an online university study recruitment database. Before participating, each eligible volunteer was required to sign the personal information and informed consent statement that was approved by the Nipissing University Research Ethics Board (14-07-04).

### Instrumentation

**Fig. 1:**
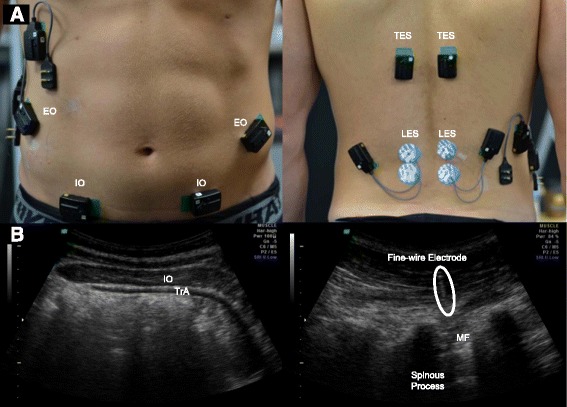
**a** Experimental setup for surface EMG: external oblique (EO), internal oblique (IO), thoracic erector spinae (TES) and lumbar erector spinae (LES). **b** Ultrasound images of the muscles of interest for indwelling EMG: internal oblique (IO), transversus abdominis (TrA), and deep multifidus (MF)

Surface EMG data were recorded at 2000Hz from four muscles bilaterally (Fig. 1a): thoracic and lumbar erector spinae (TES and LES), and internal and external oblique (IO and EO) (Trigno, Delsys Inc., USA). Prior to application of surface EMG sensors, all locations were shaved and cleaned with alcohol to ensure low impedance. Fine wire EMG was recorded synchronously from three muscles unilaterally (right side): IO, TrA, and the deep fibers of multifidus (MF). All indwelling electrodes were inserted under the guidance of ultrasound imaging (USI) (Voluson i, GE Health Care, UK) to ensure correct positioning (Fig. 1b). Kinematic data were collected concurrently at 50 Hz from 12.7 mm reflective markers (B&L Engineering, Santa Ana, CA, USA) placed over key body landmarks to track 3D whole-body motion using 13 motion capture cameras (Oqus 400+, Qualisys, Sweden) (see Additional file 1, which lists all tracking and calibration markers).

### Protocol

Following instrumentation, participants performed three maximum voluntary isometric contractions (MVICs) against manual resistance for the abdominal muscles (upper trunk flexion, upper trunk flexion combined with left and right twisting, and upper trunk flexion combined with left and right lateral bending while lying supine) and three MVICs against manual resistance for the back muscles (upper trunk extension, upper trunk extension with left and right twisting, and upper trunk flexion with left and right bending while lying prone) [20].

**Fig. 2:**
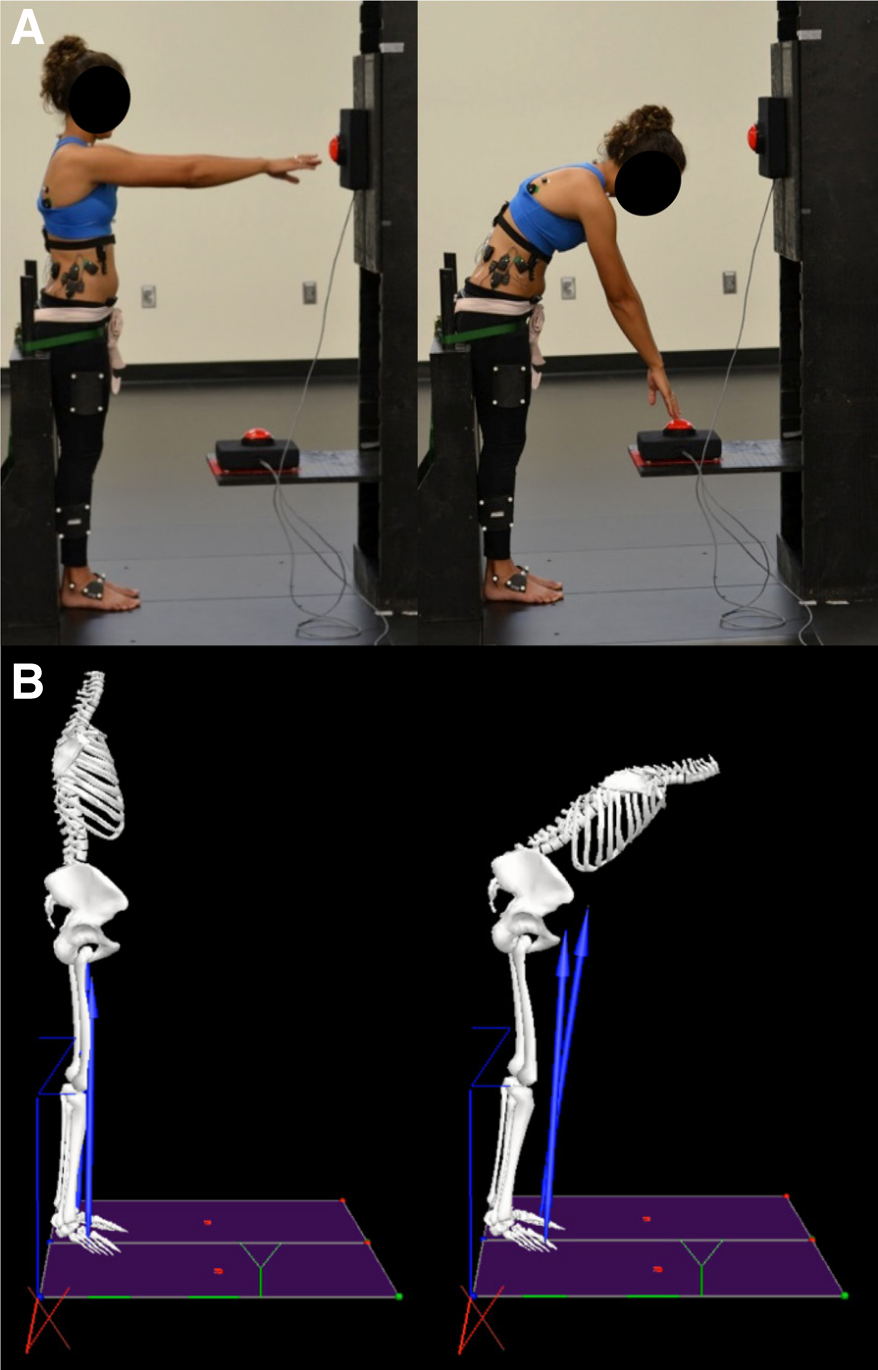
**a** Experimental setup and task requirements. **b** Visual-3D model while performing experimental task

Each participant then performed two trials of 35 cycles of repetitive unloaded spine flexion with a constrained pelvis to the beat of a metronome at a rate of 15 cycles/min [16] (Fig. 2a). This number of cycles was chosen as it provides sufficient data to obtain accurate dynamic stability estimates [21], and the rate was chosen as it has been found to be the preferred movement rate in several studies [16, 21]. Within each cycle, participants were required to touch two targets with their hands extended in front of them: the top target was located in front of them at shoulder height in the mid-sagittal plane so that it could be reached when standing upright with the arms extended, while the second target was located in the mid-sagittal plane, 50 cm anterior to the knee when participants were standing upright with their hips and knees extended [16, 22, 23] (Fig. 2a).

Between trials, participants were instructed by a Registered Physiotherapist with post-graduate training and experience in USI on how to perform the ADIM in a standing position, and USI was used as biofeedback to ensure successful contraction of the TrA. To improve the external validity of this study, the training of the ADIM closely mimicked how the ADIM would be taught in clinical practice. Participants were verbally cued to slowly draw their lower abdominal wall towards their spine. By visual inspection and palpation, the physiotherapist ensured that the participant had no posterior rotation of their pelvis and did not hold their breath during the maneuver [24]. A successful contraction (ADIM) was defined as a visible increase in thickness of the TrA on USI prior to any observed thickness changes to the IO or EO [25]. Participants were then instructed how to perform the ADIM at the onset of each repetitive movement cycle and to hold the contraction throughout the entire range of motion. Participants released their ADIM when they returned to the initial stance position and were instructed to contract their TrA at the start of every cycle. The ADIM was practiced with the movement cycle while the physiotherapist manually palpated the contraction to ensure the participant was able to hold the contraction throughout the entire movement. Once the participant could perform the activity correctly and consistently, testing began.

### Data processing & analyses

Three-dimensional whole-body motion data were processed in Visual-3D (C-Motion Inc., USA), but only 3D lumbar spine kinematics were analyzed here (Fig. 2b). EMG signals were processed by first removing the DC offset by subtracting the mean of the entire signal from each data point. Next, EMG data were bandpass filtered between 20 and 450Hz, full-wave rectified, and then linear enveloped using a second order, dual-pass Butterworth filter with a low-pass frequency cutoff of 2.5Hz [16]. EMG signals obtained during testing were then normalized to the highest smoothed amplitude obtained during any of the MVICs performed for each muscle group, after ensuring there were no non-physiological spikes in the data. Mean and peak normalized EMG amplitudes were then calculated for each muscle during each cycle and data from all cycles were used to calculate an average peak and average mean EMG signal for each participant and trial (pre/post training). Using the Euclidean norm of the 3D lumbar spine angles, local dynamic spine stability was calculated using the maximum finite-time Lyapunov exponent (λ_max_) method, which is described in more detail in a previous publication [16].

Local dynamic stability values under both baseline and trained trials were normally distributed according to Shapiro-Wilk testing and, therefore, values were compared across trials using repeated-measures ANOVA in SPSS 22 (IBM Corporation, Armonk, NY, USA). Conversely, the average peak and average mean EMG amplitudes were not normally distributed for many muscles and, therefore, were compared between baseline and trained trials using Friedman’s rank test. In all cases alpha was set to 0.05. To explore the relationship between changes in muscle activation and changes in spine stability, the absolute changes in both peak and mean muscle activation between baseline and trained trials (trained %MVIC—baseline %MVIC) were determined for all muscles (these data were normally distributed), and then added as covariates into the repeated-measures ANOVAs. A stepwise removal approach was used whereby interactions between the main effect (training) and covariates were removed if the p-value was greater than 0.2, and the covariates themselves were removed if their p-values were greater than 0.2 and they did not interact significantly with the main effect of training. This was done sequentially until all remaining interactions between the covariates and the main effects, and all remaining covariates had significance levels (p-values) less than 0.2. Moreover, to cross-validate the ANCOVA model results, Pearson’s correlations were computed between the changes in both peak and mean muscle activation for all muscles and the changes in local dynamic spine stability.

## Results

**Fig. 3:**
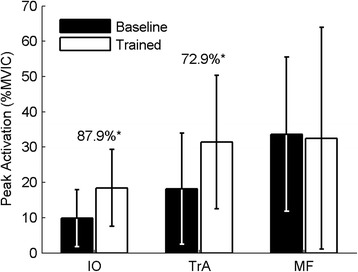
Peak indwelling EMG results. * = significance at *p* < 0.002. Numbers indicate percentage change between the baseline and trained trials. Error bars indicate standard deviations

**Fig. 4:**
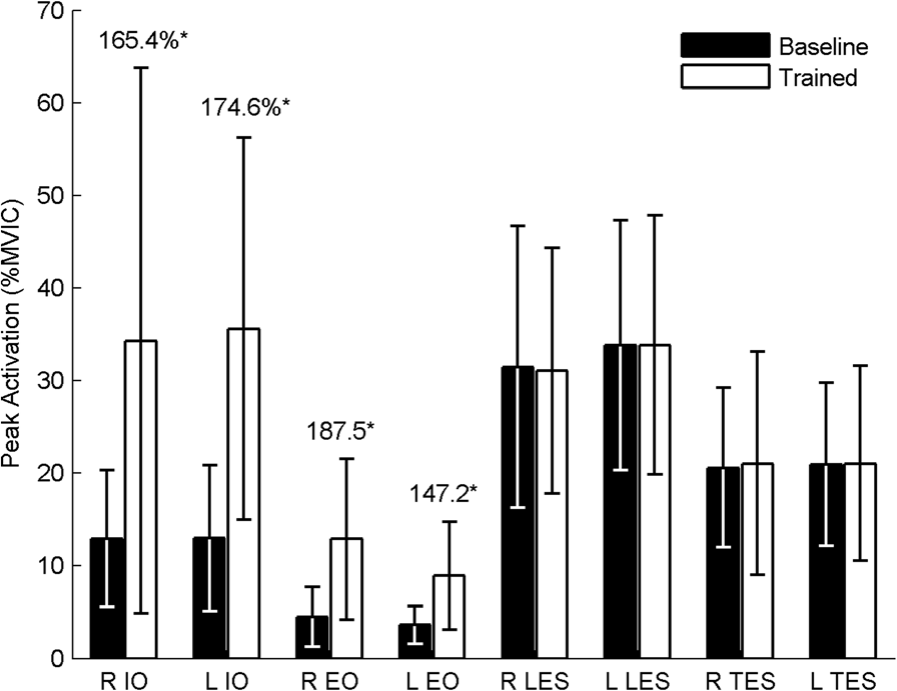
Peak surface EMG results. * = significance at *p* < 0.002. Numbers indicate percentage change between the baseline and trained trials. Error bars indicate standard deviations. R= right, L= left

**Fig. 5:**
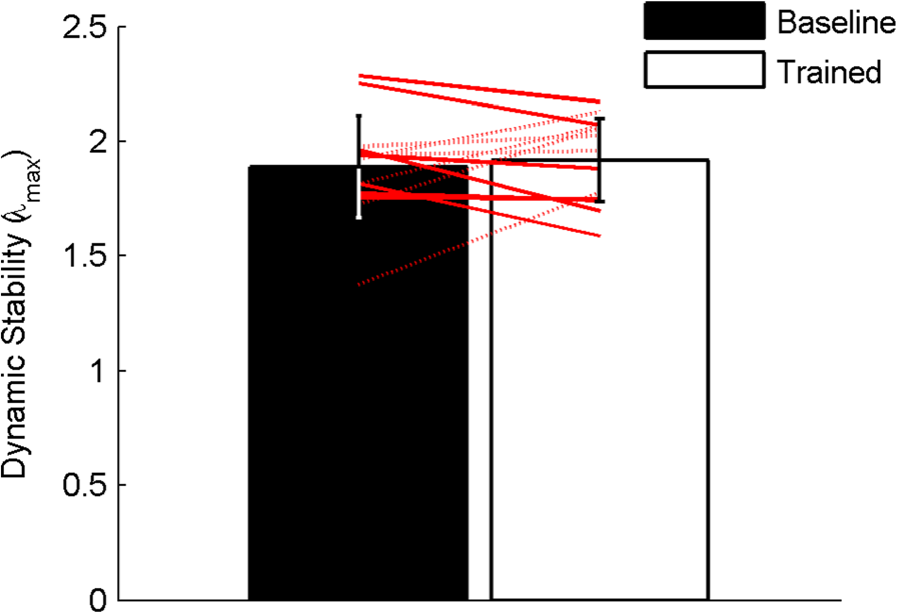
Dynamic Stability (λ_max_) results. Red lines are shown for individual participant responses. Solid lines = increased stability. Dashed lines = decreased stability. Error bars indicate standard deviations

**Fig. 6:**
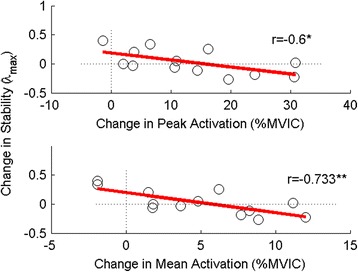
Correlation between change in stability and change in peak TrA amplitude (top) and between change in stability and change in mean TrA amplitude (bottom) * = significance at *p* < 0.05, ** = significance at *p* < 0.01

The ANOVA results for both mean and peak EMG activation showed the same trends and consequently, only peak EMG results are reported. Following the ADIM training, there were significantly greater peak levels of activation in all of the tested abdominal muscles (IO, EO, and TrA) during movement (*p* < 0.05), but no significant increases in peak activation of the back muscles (MF, LES, or TES; *p* > 0.05) (Figs. 3 and 4). There was no significant change in the local dynamic stability of the spine (λ_max_) during the task following training (*p* = 0.633) (Fig. 5). However, after adding the changes in EMG activation between baseline and trained trials (ΔEMG) for all tested muscles as covariates into the repeated-measures ANOVAs, in both cases (peak and mean), after stepwise removal of interactions with covariates and covariates themselves with *p* > 0.2, only the change in TrA activation remained. Further, with the change in TrA activation included as a covariate, a significant main effect of training on λ_max_ emerged (*p* = 0.04 and 0.01 for the change in peak and change in mean EMG activation respectively). This moderating effect of change in activation (peak or mean) on changes in λ_max_ was only true for TrA and not for any other muscle. There was also a significant interaction between the training and the change in peak and mean TrA activation (*p* = 0.030 and 0.004, respectively), whereby those with higher increases in TrA activation had lower λ_max_ after training, and those with smaller increases in TrA actually had detrimental effects on their stability (higher λ_max_). Concordantly, Fig. 6 shows that there was a significant negative correlation between the changes in peak (*r* = -0.6, *p* = 0.030) and mean (*r* = -0.733, *p* = 0.004) TrA activation and the change in λ_max_ from baseline to trained trials. No other significant findings were present when changes in EMG activation of the other tested muscles and change in λ_max_ were tested through Pearson’s correlations.

## Discussion

The overall findings from this work suggest that training individuals to perform an ADIM in standing using USI biofeedback and subsequently instructing them to perform the ADIM during a repetitive movement task does not provide group-level benefits in dynamic spine stability (i.e. there is no overall main effect of training on stability). However, this work also provides evidence that changes in TrA activation with training may positively moderate changes in dynamic spine stability (i.e. those who were most successful in improving TrA activation showed the greatest improvements in stability with training). Interestingly, this work also suggests the converse that the ADIM may be detrimental in those participants who are not successful in actively increasing the activation in their TrA (i.e. if certain participants focus on the ADIM but do not successfully increase their TrA activation, their stability may decrease).

The lack of significant group effects in this study agree with a previous study that found little benefit to using the ADIM for increasing mechanical spine stability [13]. The EMG findings support our hypothesis that training individuals to perform an ADIM would increase their activation of TrA during the repetitive task without concurrent co-activation of the posterior trunk muscles; however, similar to a previous study [13], participants did not activate TrA in isolation; they also increased activation of IO and EO during the repetitive task. Although there was no co-contraction/bracing across the lumbar spine (i.e. MF activation did not change between trained and untrained conditions), selective activation of the TrA in isolation was not achieved and this is consistent with the dynamic task demands. Previous research has found that isolating the TrA is possible in a static supine position [26]. However, due to the orientation of the muscular layers of the deep abdominals and their interlinking connective tissues, force generation in one layer will have a direct effect on the muscles adjacent to it during movement [27, 28]. This finding is important as it supports previous literature that has found it may not be possible or practical to isolate the TrA during dynamic tasks and refutes the clinical idea that isolated TrA contractions are achievable and may be beneficial to symptomatic individuals.

Although as a group, our sample did not increase spine stability following the ADIM training, when the change in TrA activation from the baseline to the trained state was added as a covariate into the repeated-measures ANOVA, the main effect of training became significant for changes in both peak (*p* = 0.04) and mean (*p* = 0.01) TrA activation. Furthermore, there was also a significant interaction between the training and the change in both the peak (*p* = 0.03) and mean (*p* = 0.004) TrA activation, whereby those with higher increases in TrA activation demonstrated lower λ_max,_ and thus improved stability. Consistent with this interaction, we found a significant negative correlation between the change in TrA activation and the change in λ_max_ from baseline to trained trials, which also showed that as activation of the TrA increased, the λ_max_ exponent decreased.

Other than TrA, no changes in the activation of other abdominal or lumbar muscles were significant when added as covariates into the repeated-measures ANOVA, and there were no significant correlations between change in stability and change in activation of any of the other muscles tested. This finding may suggest that activation of the TrA has a role in the stabilization of the spine in at least some individuals. This finding may also indicate that some participants were more successful than others at performing the ADIM, and those who were more successful may have indeed increased their stability. Given that the training session was brief and occurred only once, some of the participants may not have had enough practice to adequately master the ADIM to the point of increasing stability. Although not reported, abdominal muscle activation variability was significantly greater during the trained trial, and thus the non-familiarity of performing an ADIM during movement may in part be the reason we did not see an increase in stability.

There are several potential reasons why stability may have decreased in those individuals who were unsuccessful in increasing their TrA activation. Previous literature has shown that focusing internally on the activation of a muscle rather than externally on the task at hand can impair neuromuscular coordination and movement outcomes during an isometric plantar-flexion task [29]. It has also been shown that focusing externally on the task at hand produces more accurate and consistent movements with less error and a more efficient motor recruitment pattern [30–32]. Furthermore, performing the ADIM during a dynamic movement task may be considered a dual-task, which has been shown previously to have the ability to impair dynamic postural control [33] as well as dynamic gait stability measured using maximum finite-time Lyapunov exponents [34]. Thus, in those participants who were not able to adequately increase their TrA activation through the ADIM, an internal focus (i.e. on the performance of the ADIM rather than the task at hand (repetitive flexion)) or performing this dual-task without receiving the potential benefits of increased TrA activation may have led to impaired stability. To assess such effects, future studies should retest participants with a follow-up session after they have performed take home exercises and received supervised ADIM training sessions and re-evaluation through which it is confirmed that they perform the task consistently and appropriately.

Finally, it is also important to note that the results from this study may simply illustrate the large variability in the population, such that for some individuals the ADIM may be an effective method for increasing spine stability, while for others it may not be. The variable response to the ADIM seen in our sample may be very important clinically, as a 5-min training session may be beneficial to some individuals, but detrimental to others. A greater number of participants may have been more successful at the ADIM task if a longer training session was implemented. However, this study aimed to mirror what is typically done during one physiotherapy treatment session in order to improve the external validity of this work. Whether the significant main effect of training that was seen when change in TrA activation was added as a covariate into the repeated-measures ANOVA was due to mechanical factors (intra-abdominal pressure, fascial tension) or to the muscle activation itself remains in question and requires further investigation.

## Conclusions

The current study, which employed a single cohort pre/post-intervention protocol to examine whether instruction on the performance of the ADIM prior to a dynamic movement task alters dynamic spine stability and control, showed no main effect of training on spine stability. However, the fact that change in TrA activation was the only significant covariate that moderated spine stability suggests a possible role for the TrA in spine stability for some individuals. If this is the case, this study highlights an important clinical concept. Although the use of the ADIM as a stabilization exercise is popular in the physiotherapy management of LBP, the rationale for its use may not be supported. Rehabilitation techniques may need to be individualized rather than generalized for a specific population and performing the ADIM in isolation may not improve stability. Reliable and valid clinical evaluations may need to be developed to determine which individuals may benefit from this intervention.

### Ethics approval

Nipissing University (14-07-04).

## Additional file

Additional file 1:
**Document containing the full listing and positions of tracking and calibration markers used to determine 3D whole body kinematics.** (PDF 4 kb)

## References

[1] Panjabi MM (1992). The stabilizing system of the spine. Part I. Function, dysfunction, adaptation, and enhancement. J Spinal Disord.

[2] Cholewicki J, VanVliet JJ (2002). Relative contribution of trunk muscles to the stability of the lumbar spine during isometric exertions. Clin Biomech.

[3] Kavcic N, Grenier S, McGill SM (2004). Determining the stabilizing role of individual torso muscles during rehabilitation exercises. Spine (Phila Pa 1976).

[4] McGill SM, Grenier S, Kavcic N, Cholewicki J (2003). Coordination of muscle activity to assure stability of the lumbar spine. J Electromyogr Kinesiol.

[5] Hodges PW (1999). Is there a role for transversus abdominis in lumbo-pelvic stability?. Man Ther.

[6] Barker PJ, Guggenheimer KT, Grkovic I, Briggs CA, Jones DC, Thomas CDL, Hodges PW (2006). Effects of tensioning the lumbar fasciae on segmental stiffness during flexion and extension. Spine (Phila Pa 1976).

[7] Hodges PW, Cresswell AG, Daggfeldt K, Thorstensson A (2001). In vivo measurement of the effect of intra-abdominal pressure on the human spine. J Biomech.

[8] Hodges PW, Moseley GL, Gabrielsson A, Gandevia SC (2003). Experimental muscle pain changes feedforward postural responses of the trunk muscles. Exp Brain Res.

[9] Hodges WP, Martin Eriksson AE, Shirley D, Gandevia CS (2005). Intra-abdominal pressure increases stiffness of the lumbar spine. J Biomech.

[10] Richardson CA, Snijders CJ, Hides JA, Damen L, Pas MS, Storm J (2002). The relation between the transversus abdominis muscles, sacroiliac joint mechanics, and low back pain. Spine (Phila Pa 1976).

[11] Hodges PW, Richardson CA (1998). Delayed postural contraction of transversus abdominis in low back pain associated with movement of the lower limb. J Spinal Disord.

[12] Hodges PW, Richardson CA (1996). Inefficient muscular stabilization of the lumbar spine associated with low back pain. Spine (Phila Pa 1976).

[13] Grenier SG, McGill SM (2007). Quantification of lumbar stability by using 2 different abdominal activation strategies. Arch Phys Med Rehabil.

[14] Hodges P (2008). Transversus abdominis: a different view of the elephant. Br J Sports Med.

[15] Reeves NP, Narendra KS, Cholewicki J (2007). Spine stability: the six blind men and the elephant. Clin Biomech (Bristol, Avon).

[16] Graham RB, Oikawa LY, Ross GB (2014). Comparing the local dynamic stability of trunk movements between varsity athletes with and without non-specific low back pain. J Biomech.

[17] Reynard F, Vuadens P, Deriaz O, Terrier P (2014). Could local dynamic stability serve as an early predictor of falls in patients with moderate neurological gait disorders? A reliability and comparison study in healthy individuals and in patients with paresis of the lower extremities. PLoS One.

[18] van Schooten KS, Pijnappels M, Rispens SM, Elders PJM, Lips P, van Dieën JH (2015). Ambulatory fall-risk assessment: amount and quality of daily-life gait predict falls in older adults. J Gerontol Ser A Biol Sci Med Sci.

[19] Ross GB, Mavor M, Brown SHM, Graham RB (2015). The effects of experimentally induced low back pain on spine rotational stiffness and local dynamic stability. Ann Biomed Eng.

[20] Vera-Garcia FJ, Moreside JM, McGill SM (2010). MVC techniques to normalize trunk muscle EMG in healthy women. J Electromyogr Kinesiol.

[21] Dupeyron A, Rispens SM, Demattei C, van Dieën JH (2013). Precision of estimates of local stability of repetitive trunk movements. Eur Spine J.

[22] Granata KP, England S a (2006). Stability of dynamic trunk movement. Spine (Phila Pa 1976).

[23] Granata KP, Gottipati P (2008). Fatigue influences the dynamic stability of the torso. Ergonomics.

[24] Henry SM, Westervelt KC (2005). The use of real-time ultrasound feedback in teaching abdominal hollowing exercises to healthy subjects. J Orthop Sports Phys Ther.

[25] Hides JA, Wilson S, Stanton W, McMahon S, Keto H, McMahon K, Bryant MB, Richardson CA (2006). An MRI investigation into the function of the transversus abdominis muscle during “Drawing-In” of the abdominal wall. Spine (Phila Pa 1976).

[26] Urquhart DM, Hodges PW, Allen TJ, Story IH (2005). Abdominal muscle recruitment during a range of voluntary exercises. Man Ther.

[27] Huijing P a, Baan GC (2003). Myofascial force transmission: muscle relative position and length determine agonist and synergist muscle force. J Appl Physiol.

[28] Brown SHM, McGill SM (2009). Transmission of muscularly generated force and stiffness between layers of the rat abdominal wall. Spine (Phila Pa 1976).

[29] Lohse KR, Sherwood DE (2012). Thinking about muscles: The neuromuscular effects of attentional focus on accuracy and fatigue. Acta Psychol (Amst).

[30] Zachry T, Wulf G, Mercer J, Bezodis N (2005). Increased movement accuracy and reduced EMG activity as the result of adopting an external focus of attention. Brain Res Bull.

[31] Wulf G (2013). Attentional focus and motor learning: a review of 15 years. Int Rev Sport Exerc Psychol.

[32] Lohse KR, Sherwood DE, Healy AF (2011). Neuromuscular effects of shifting the focus of attention in a simple force production task. J Mot Behav.

[33] Donker SF, Roerdink M, Greven AJ, Beek PJ (2007). Regularity of center-of-pressure trajectories depends on the amount of attention invested in postural control. Exp Brain Res.

[34] Lamoth CJ, van Deudekom FJ, van Campen JP, Appels BA, de Vries OJ, Pijnappels M (2011). Gait stability and variability measures show effects of impaired cognition and dual tasking in frail people. J Neuroeng Rehabil.

