# Supplementary material for: The acute effects of targeted abdominal muscle activation training on spine stability and neuromuscular control
Source: J Neuroeng Rehabil. 2016 Feb 27;13:19. doi: 10.1186/s12984-016-0126-9 (PMC4769829; doi:10.1186/s12984-016-0126-9)
Supplement: Additional file 1: — Document containing the full listing and positions of tracking and calibration markers used to determine 3D whole body kinematics. (PDF 4 kb) [file 12984_2016_126_MOESM1_ESM.pdf]

Participants were outfitted with 12.7mm reflective markers (B&L Engineering, Santa Ana, CA, USA) on the following locations for tracking whole body motion: upper back (C_7, medial border of the inferior angle of the right and left scapulae, right and left acromioclavicular joints, and sternal notch); lower back (cluster over T12); pelvis (cluster over S1); right and left thigh (cluster placed laterally at the mid-point between the greater trochanter and lateral femoral epicondyle); right and left shank (cluster placed laterally at the mid-point between the lateral tibial condyle and the lateral malleolus); and right and left foot (lateral malleoli and cluster).

A second set of calibration-only reflective markers was applied during calibration trials on the following locations: xiphoid process, right and left anterior superior iliac spine, right and left posterior superior iliac spine, right and left iliac crest, right and left greater trochanter, right and left lateral femoral epicondyle, right and left medial femoral epicondyle, right and left tibial tubercle, right and left medial malleolus, right and left calcaneus, right and left first metatarsal (distal aspect), and right and left fifth metatarsal (distal aspect).

Although whole-body kinematic data were collected and analyzed, only low back motion data (lumbar spine vs. pelvis) are reported in the current study.
